# Ascites management by cell-free concentrated ascites reinfusion therapy during recovery from drug-induced acute liver injury: a case report

**DOI:** 10.1186/s13256-020-02507-5

**Published:** 2020-10-14

**Authors:** Koya Yasuda, Mea Asou, Tomohiko Asakawa, Makoto Araki

**Affiliations:** Department of Internal Medicine, Suwa Central Hospital, 4300 Tamagawa, Chino-shi, Nagano-ken, 391-8503 Japan

**Keywords:** drug-induced liver injury, cell-free concentrated ascites reinfusion therapy, warfarin, diltiazem

## Abstract

**Background:**

The symptoms of drug-induced hepatic injury are manifold; however, the presence of ascites indicates a severe disease condition. The rapid accumulation of ascites is distressing and requires palliative treatment. Because many cases are addressed by repeated large-volume paracentesis, often resulting in impairment due to protein and electrolyte loss, a different approach is required.

**Case presentation:**

A 61-year-old Japanese man on maintenance dialysis was admitted to our hospital with acute liver injury. Our patient was diagnosed as having drug-induced liver injury due to warfarin or diltiazem, which started immediately after coronary artery bypass grafting 7 months previously. One month after admission, our patient’s hepatic encephalopathy remained grade 1 and his prothrombin time international normalized ratio was maintained at < 1.5. However, the liver was markedly atrophied with massive ascites. Although liver transplantation was desired, he was considered unfit for transplantation because of his renal and cardiac complications. Therefore, we devised a strategy to manage the massive ascites with cell-free concentrated ascites reinfusion therapy while awaiting liver regeneration. At first, cell-free concentrated ascites reinfusion therapy was required frequently because ascites accumulated rapidly. But the fluid retention interval was gradually extended as intended, and cell-free concentrated ascites reinfusion therapy was withdrawn after 8 months. During that time, the size of his liver increased from 1419 cm^3^ to 1587 cm^3^ on computed tomography.

**Conclusions:**

Cell-free concentrated ascites reinfusion therapy is an apheresis therapy in which ascites are collected aseptically by paracentesis, concentrated, and then reinfused intravenously. This treatment has the advantage of preserving nutrition by reusing the fluid. Previously, cell-free concentrated ascites reinfusion therapy was used only for the management of ascites in patients with cirrhosis or carcinomatous peritonitis. This case suggests that palliation and maintenance of nutritional status with cell-free concentrated ascites reinfusion therapy may be useful as an adjunct to liver regeneration in drug-induced hepatic injury.

## Background

Drug-induced liver injury (DILI) is the most common cause of acute liver failure. The incidence is 14 to 19 cases per 100,000 persons, of which 30% are associated with jaundice [1]. In recent years, the incidence of DILI has been increasing annually, with a steady rise in the number of clinical agents implicated.

Cardiovascular agents account for 10% of DILI and are the third most common cause after antibiotics and supplements [2]. Acute liver injury caused by DILI often requires liver transplantation. However, if there are many cardiovascular complications, transplantation may be contraindicated because of the high risk. In such cases, conservative treatment is the only way to improve liver function.

In this case, liver regeneration was confirmed 8 months after adjuvant therapy for drug-induced acute liver injury. The characteristic feature of this case is that cell-free concentrated ascites reinfusion therapy (CART) was performed for refractory massive ascites to relieve the pain without lowering the nutritional state.

## Case presentation

**Fig. 1:**
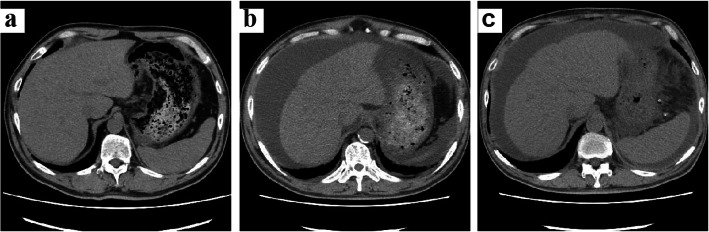
Abdominal computed tomography (CT). **a** (Day 0): the liver is enlarged. The biliary system obstruction mechanism is not recognized. The liver volume measured by CT is 1973 cm^3^. **b** (Day 37): the liver atrophied with massive ascites. The liver volume is 1419 cm^3^. **c** (Day 284): the liver is regenerating and growing. Ascites are reduced. The liver volume is 1587 cm^3^. The number of days from the date of hospitalization to the date of examination

A 61-year-old Japanese man on maintenance dialysis was admitted to our hospital with jaundice and hepatobiliary enzymes abnormality. Our patient had diabetes mellitus, and hemodialysis had been started 3 years earlier for diabetic nephropathy. He had a history of myocardial infarction and had undergone coronary artery bypass grafting (CABG) 7 months earlier for unstable angina. He had never consumed alcohol or traveled abroad. He was mildly obese and his body mass index was 25.1 kg/m^2^. Except for mild jaundice in his skin and palpebral conjunctiva, there were no major physical abnormalities. The laboratory findings suggested a liver and biliary tract disease (Table 1). The coagulation profile was slightly prolonged because our patient was taking warfarin. An abdominal computed tomography (CT) scan showed swelling of his liver and edema of the periportal space, without biliary obstruction (Fig. 1a).

**Table 1:** Laboratory findings on admission

		Unit			Unit
WBC	6820	/μL	IgG	1479	mg/dL
Hb	10.9	g/dL	IgA	253	mg/dL
Plt	16.5	10^4^/uL	IgM	28	mg/dL
PT-INR	2.92		IgE	273	IU/mL
APTT	64	sec			
TP	6.3	g/dL	IgM HAV Ab	(-)	
Alb	3.3	g/dL	HBs Ag	(-)	
AST	258	IU/L	IgM HBc Ab	(-)	
ALT	261	IU/L	HBs Ab	(-)	
LDH	318	IU/L	HCV Ab	(-)	
CPK	77	IU/L	HCV RNA	(-)	
ALP	606	IU/L	IgA HEV Ab	(-)	
γ-GTP	443	IU/L	CMV IgM Ab	(-)	
T-Bil	5.25	mg/dL	CMV IgG Ab	(+)	
D-Bil	4.3	mg/dL	EBV IgM Ab	(-)	
BUN	15.7	mg/dL	EBV IgG Ab	(-)	
Cr	5.68	mg/dL	EBV EBNA	(+)	
Na	139.2	mEq/L			
K	3.7	mEq/L	ANA	(-)	
Cl	100	mEq/L			
CRP	2.99	mg/dL			

*Ab* antibody, *Ag* antigen, *HAV* Hepatitis A virus, *HBs* hepatitis B surface, *HBc* hepatitis B core antigen, *HBs* hepatitis B surface antigen, *HCV* Hepatitis C virus, *HEV* Hepatitis E virus, *CMV* citomegalovirus, *EBV* Epstein-Barr virus, *EBNA* EBV nuclear antigen, *ANA* anti-nuclear antibody

**Fig. 2:**
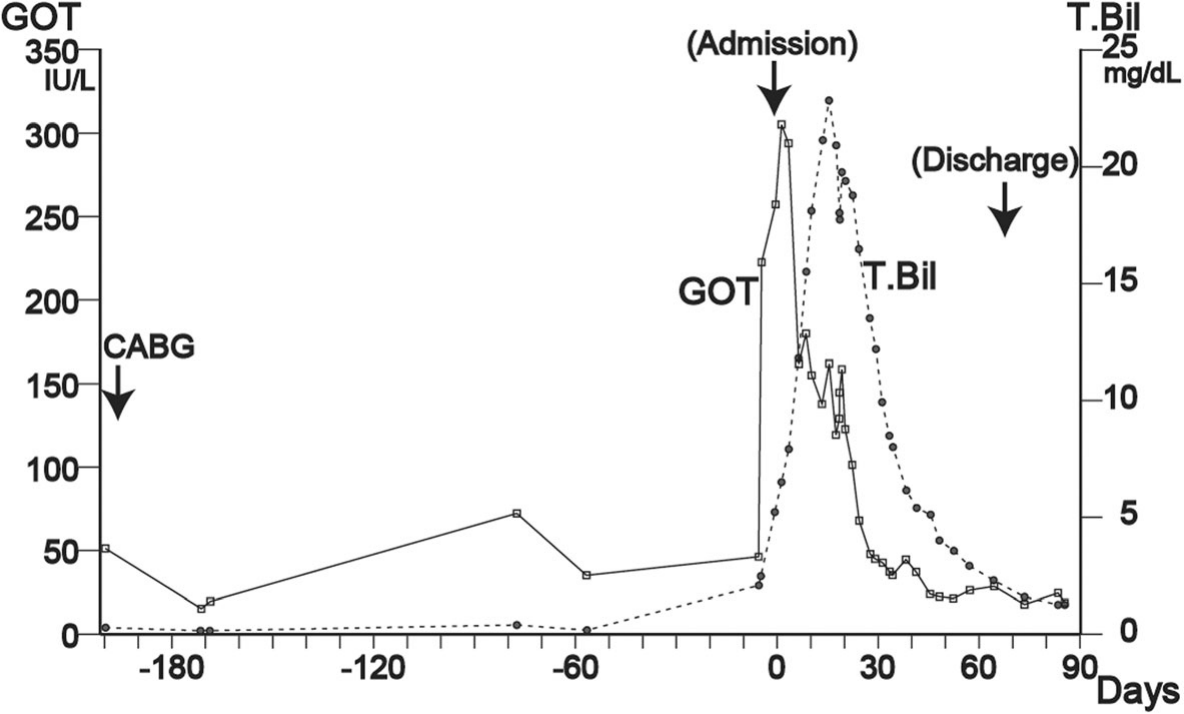
Transition of glutamic oxaloacetic transaminase and total bilirubin. Warfarin and diltiazem were started after coronary artery bypass grafting surgery. Other drugs were started more than 2 years previously. All drugs were stopped immediately after admission. Maximum glutamic oxaloacetic transaminase of 306 IU/ml (Day 2) and total bilirubin of 22.8 mg/dl (Day 17).

**Fig. 3:**
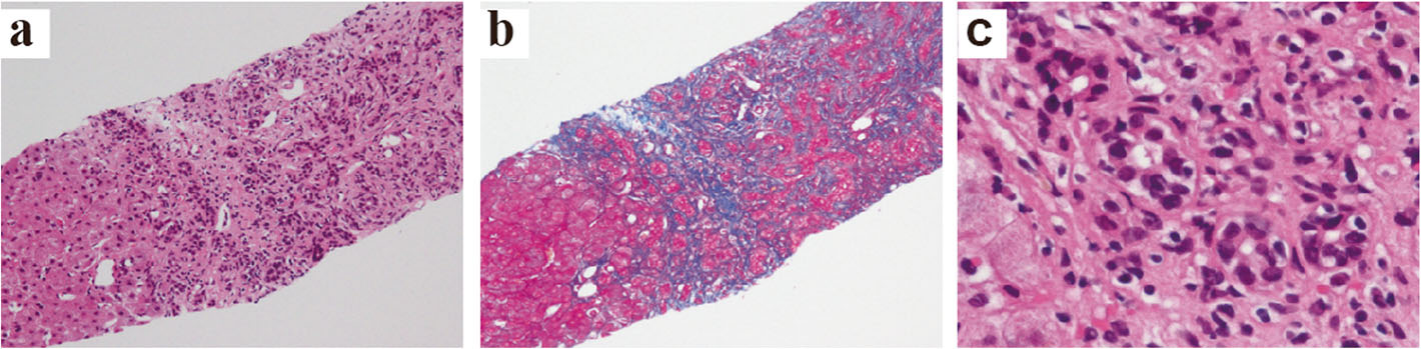
Liver biopsy (Day 46). **a,b** Fibrotic enlargement and piecemeal necrosis of bile canaliculi were observed in the portal area. But lymphocytic infiltration and hepatocellular necrosis of the hepatic lobuleis were rarely observed. (**a**: hematoxylin-eosin stain, **b**: azan stain, ×40). **c** Proliferation of bile canaliculi (hematoxylin-eosin stain, ×400)

Viruses and autoimmune diseases were considered as the possible cause of his liver disease, and various tests were performed, but no abnormalities suggesting the cause were found (Table 1). Our patient had been taking aspirin, prasugrel, carvedilol, nicorandil, linagliptin, rosuvastatin, esomeprazole, alfacalcidol, and precipitated calcium carbonate for more than 2 years. Warfarin and diltiazem had been started after his CABG 7 months earlier. Considering the possibility of DILI, all drugs were discontinued from the first day of hospitalization, but his liver function continued to deteriorate, reaching the maximum glutamic oxaloacetic transaminase (GOT) of 306 IU/ml (Day 2) and total bilirubin of 22.8 mg/dl (Day 17). Subsequently, his blood test results gradually improved, but ascites appeared and his abdomen became painfully distended (Fig. 2). CT showed marked atrophy of his liver with massive ascites (Fig. 1b). We made a diagnosis of DILI by warfarin or diltiazem, by exclusion based on blood test results and clinical course. On Day 46, a liver biopsy was performed. Fibrotic enlargement, piecemeal necrosis, and proliferation of bile canaliculi were observed in the portal area. On the other hand, within the hepatic lobule, very slight lymphocytic infiltration, and a very small amount of hepatocellular necrosis were observed. These findings were consistent with mixed-type DILI, mainly cholestatic injury (Fig. 3).

On Day 8, our patient’s Model for End-Stage Liver Disease (MELD) score was 32 (MELD score: total bilirubin - 11.9 mg/dl, prothrombin time-international normalized ratio [PT-INR] - 1.3, dialysis patient), and symptoms equivalent to grade 1 hepatic encephalopathy (insomnia and depression) were observed. Therefore, we asked to perform a liver transplantation at a university hospital, but he was refused because of his many complications. We considered peritoneovenous shunt or transjugular intrahepatic portosystemic stent shunts (TIPS) because of a persistent grade 3 ascites as defined by the International Ascites Club. However, we could not obtain consent because of the risk of infection and complications. Therefore, we decided to perform cell-free concentrated ascites reinfusion therapy (CART) on Day 45. CART is a type of apheresis therapy in which ascites undergo sterile filtration and are concentrated, containing albumin and globulin, and reinfused into the patient. His pain was relieved on removing 8 l of ascitic fluid. His blood pressure remained stable during CART. There was 56.6 g of albumin collected from the ascites, of which 49.4 g (87.2%) was reinjected intravenously.

**Fig. 4:**
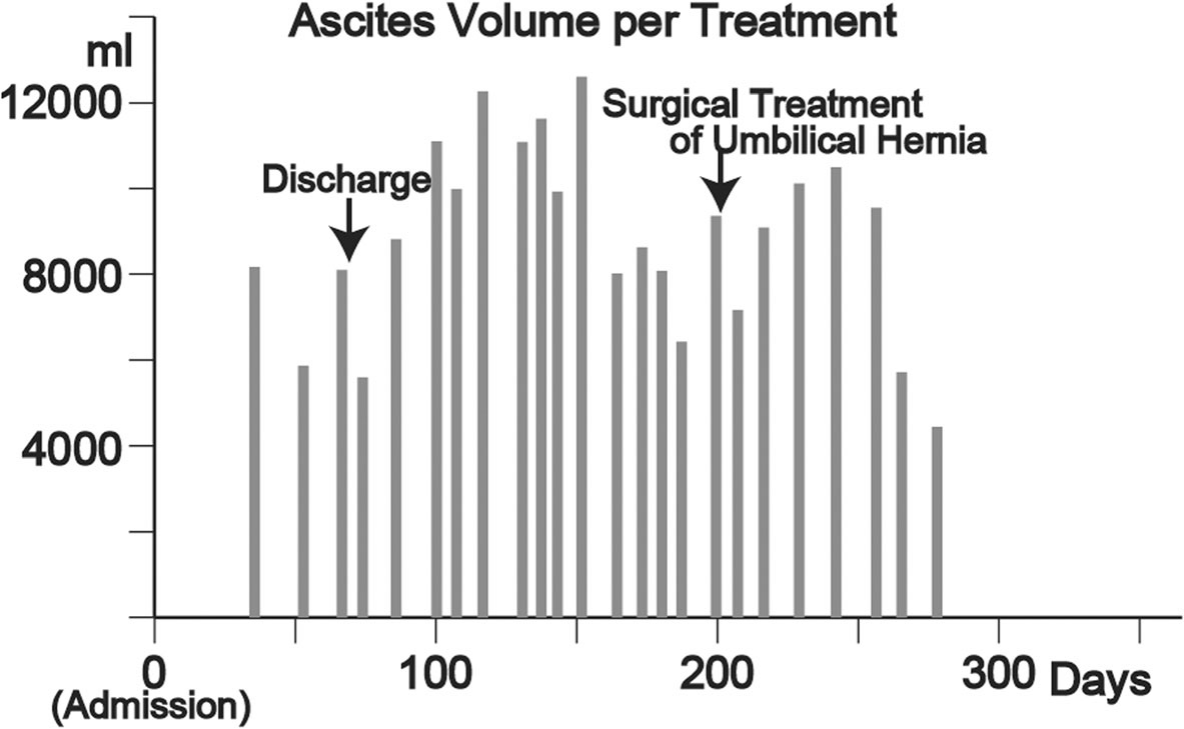
Ascites volume during cell-free concentrated ascites reinfusion therapy (CART). CART was started on Day 45 and ended on Day 274. Our patient developed an umbilical hernia on Day 198, and enterectomy was performed. In 24 CART sessions, an average of 8.7 ± 2.2 l of ascites fluid was concentrated to 9.2 ± 1.5%, and 65.4 ± 22.3 g of albumin (average recovery rate 72.5 ± 8.6%) was reinjected intravenously. The number of days from the date of hospitalization to the date of examination

Although the initial schedule was once every 2 weeks, the rate of ascitic fluid accumulation was rapid and our patient was in great distress, so CART was administered once a week (Fig. 4). Our patient was discharged on Day 67 because his general condition was stabilized by CART. After discharge, hemodialysis was continued and CART was performed according to his symptoms. Our patient developed an umbilical hernia 131 days after discharge, and enterectomy was performed, but no perioperative problems occurred. Albumin was 2.1–2.5 g/dl from the onset of DILI until surgery and remained above 2.5 g/dl thereafter.

He was finally weaned from CART therapy 206 days after discharge. In 24 CART sessions, an average of 8.7 ± 2.2 l of ascitic fluid was concentrated to 9.2 ± 1.5%, and 65.4 ± 22.3 g of albumin (average recovery rate 72.5 ± 8.6%. [Recovery rate: an indicator of how much albumin could be concentrated without loss.]) was reinjected intravenously. His abdominal CT scan showed that his liver had regenerated and enlarged (Fig. 1c), which was considered to be the cause of the decreased ascites. More than 1 year later, his albumin was > 3 g/dl, PT-INR was < 1.3, total bilirubin remained within the normal range, and dialysis therapy was continued without any problem.

## Discussion

In this case, a dialysis patient with severe DILI was treated with CART a total of 24 times, and liver regeneration was eventually achieved. In the case of severe DILI, liver transplantation is often considered, but this patient was judged to be unsuitable for transplantation because of the number of complications. Therefore, adjuvant therapy was performed with the expectation of spontaneous recovery, and fortunately, his liver function improved. This case is characterized by DILI caused by warfarin or diltiazem, which was started 7 months earlier, and our patient’s pain was alleviated and his serum albumin level maintained by CART.

Many drugs that cause liver damage have been reported and this can occur in dialysis patients [3]. Intrinsic hepatotoxins and idiosyncratic reactions (nonimmune type or immune-mediated type) are assumed as mechanisms of DILI, but there are many cases in which the cause is not clear. Therefore, a classification based on the type of liver damage - hepatocellular, cholestatic, or mixed - is often used. Diagnosis is based on history and exclusion by blood tests, but causality is difficult to establish. For this reason, it is also important whether the suspect drug has been reported or not. Several scoring systems have been tried, including the Drug-Induced Liver Injury Network [4], but none have been generalized.

Treatment of DILI focuses on stopping the suspected drug. No liver-supporting agents have been identified, and steroids are considered only if they are allergic. Liver transplantation is preferred when the likelihood of spontaneous recovery from liver damage is low. One prognostic predictor of acute liver injury is the MELD score. Although the MELD score is a model for predicting mortality in patients with chronic liver disease, it is also used to predict survival in patients with acute liver failure [5]. In this case, the MELD score was 32 and liver transplantation was considered. However, since CABG had been performed 7 months earlier, and there were many complications such as diabetes mellitus and maintenance dialysis for diabetic nephropathy, transplantation was considered unsuitable.

Since most of the oral medicines had been used for more than 2 years, warfarin and diltiazem, which were started 7 months earlier, were considered as the causative agents. Warfarin therapy causes only approximately 0.8–1.2% risk of transaminase elevation > 3 upper limit of normal [6]. Liver damage with coumarin derivatives other than warfarin is more common; for example, fenprocumone has a high incidence of liver damage (2%) with (sub-)acute liver failure occurring in 0.2% [7]. Warfarin and fenprocumone are known to be cross-reactive, suggesting that the mechanisms of liver damage are the same [7]. The liver damage of fenprocumone occurs after a long incubation period of several months to several years after administration, and the course of this case is similar to that of fenprocumone. Warfarin-induced liver damage is usually cholestatic injury, although hepatocellular and mixed forms have been reported [8]. The cause of warfarin’s liver damage is still unknown, but re-administration of warfarin rapidly induces liver damage, suggesting that immunologic mechanisms are involved. Diltiazem-induced liver injury is usually mild and transient, and acute liver injury is rare [9]. This is thought to be a feature of the class of calcium antagonists themselves. Diltiazem-induced liver injury is characterized by hypersensitivity, such as fever, rash, and eosinophilia. Furthermore, its incubation period is as short as 3 to 14 days. Jaundice is often absent or mild. The pattern of liver damage can be cholestatic or hepatocellular forms. The mechanism of liver damage is unknown, but its clinical characteristics suggest that it may be due to hypersensitivity. There is a high possibility that this case of DILI was caused by warfarin, because the incubation period until the hepatic disorder appeared after the administration of the drug was more than half a year, it was a serious acute hepatic disorder, and the pathology was a mixed-type disorder mainly caused by cholestasis.

Management of non-hepatic complications is also important in the treatment of acute liver injury, and ascites is one of the major complications. Other complications, such as hepatic encephalopathy, bleeding tendency, and infection, were not significant in this case. It is also known that ascites causes hepatorenal syndrome, hyponatremia, and hypotension. However, since this case was under the special environment of maintenance dialysis, there was little such effect. Ascites is usually controlled with diet and drug volume management. However, it is known that 10% of patients with cirrhosis do not respond to such volume adjustments [10, 11] In such cases, abdominal paracentesis is the mainstay of treatment. Since the abdominocentesis causes hypotension, hypoproteinemia, and prerenal renal insufficiency, the drainage quantity is often limited to about 3 liters. As a result, symptomatic improvement remains mild, small amounts of reentrant ascites drain frequently, and the patient gradually becomes debilitated. A modified version, repeated large-volume paracentesis (LVP), is rapid and effective, but to prevent hypotension, replacement of 6–8 g albumin per liter is required [12]. Another method of managing ascites is peritoneovenous shunt, but it has not spread widely due to the risk of infection. TIPS has also been attempted to reduce portal pressure and the production of ascites, but it is not a standard treatment because of the difficulty of the procedure and complications.

The most important therapeutic feature of this case is that CART was performed for refractory massive ascites of acute liver injury with a reversible component. CART is an apheresis therapy in which cells, bacteria, and other unwanted components are removed from ascites using a filtration membrane, and necessary substances, such as protein, are concentrated sterilely, then the concentrated ascites is reinfused intravenously. The ability to maintain nutritional status is the greatest attraction of CART. Partial resection of the small intestine due to umbilical hernia was performed, but no anastomotic leakage occurred after surgery in this case. Although several methods of CART have been developed, [13–15] we chose the KM-CART developed by Matsuzaki *et al.* [14]. In this method, an average of 6.4 l of cancerous ascites was concentrated to 0.8 l in 57 minutes, with reported recovery rates of 71.1% albumin and 57.6% globulin, and our data were similar. In this method, filtration of ascites and reinfusion of concentrated solution can be performed in a short time, and the recovery rate of albumin is high. Therefore, even if all ascites are drained, dangerous complications such as shock are unlikely to occur. Side effects included a slight increase in body temperature after reinfusion, but no clinically significant adverse events occurred. Especially, since this case was a dialysis patient, CART was performed on a non-dialysis day, but cardiac failure was not caused by reinfusion. The 65.4 g of albumin we recovered is equivalent to 260 ml of 25% albumin solution. The cost of CART is a needle used for paracentesis of ascites, the back for the sterile collection of ascites, and the separation membrane used in the concentration process. Depending on the price of the membranes, albumin solutions may be cheaper in Japan in terms of price. However, CART has many advantages that are not reflected in the price, such as no risk of infection peculiar to blood products due to the use of the patient’s own ascites, and recovery of globulin as well as albumin.

## Conclusions

Acute drug-induced liver injury caused by warfarin or diltiazem in a maintenance hemodialysis patient was successfully treated with CART. CART can not only relieve pain but also maintain nutritional status and help regenerate the liver. These results suggest that CART is useful as an adjunctive therapy for refractory ascites in acute liver injury with a reversible component.

## Data Availability

All of the data and materials will be available upon request to the corresponding author.
